# Supplementary figures and images for: GWAS of adventitious root formation in roses identifies a putative phosphoinositide phosphatase (SAC9) for marker-assisted selection
Source: PLoS One. 2023 Aug 18;18(8):e0287452. doi: 10.1371/journal.pone.0287452 (PMC10437954; doi:10.1371/journal.pone.0287452)

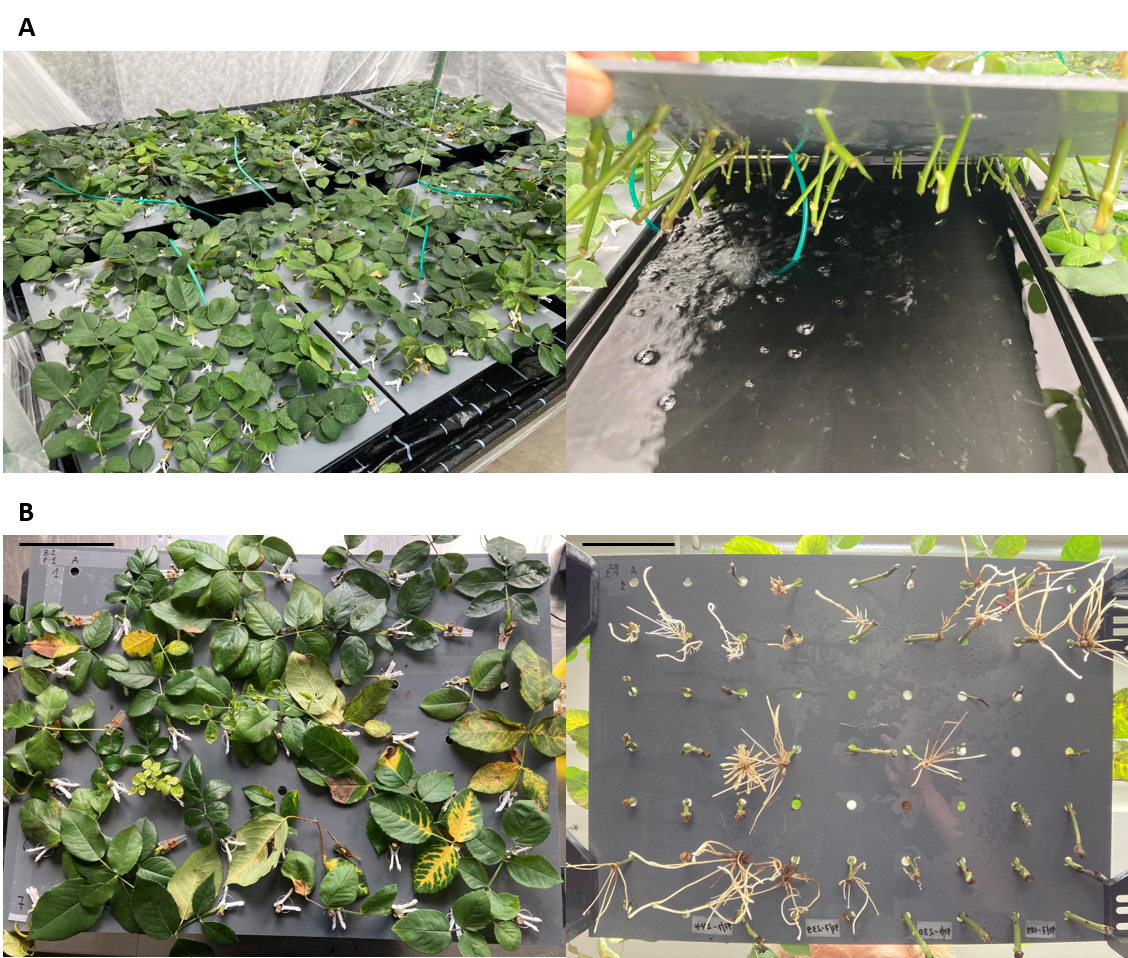

Supplement: S1 Fig — (A) Set-up of plastic plates with holes to hold rose cuttings placed on black trays filled with tap water (for two weeks) or fertiliser solution. (B) Top and bottom views of cuttings after 6 weeks of cultivation. Scale bars indicate 10 cm. (TIF) [file pone.0287452.s001.tif]

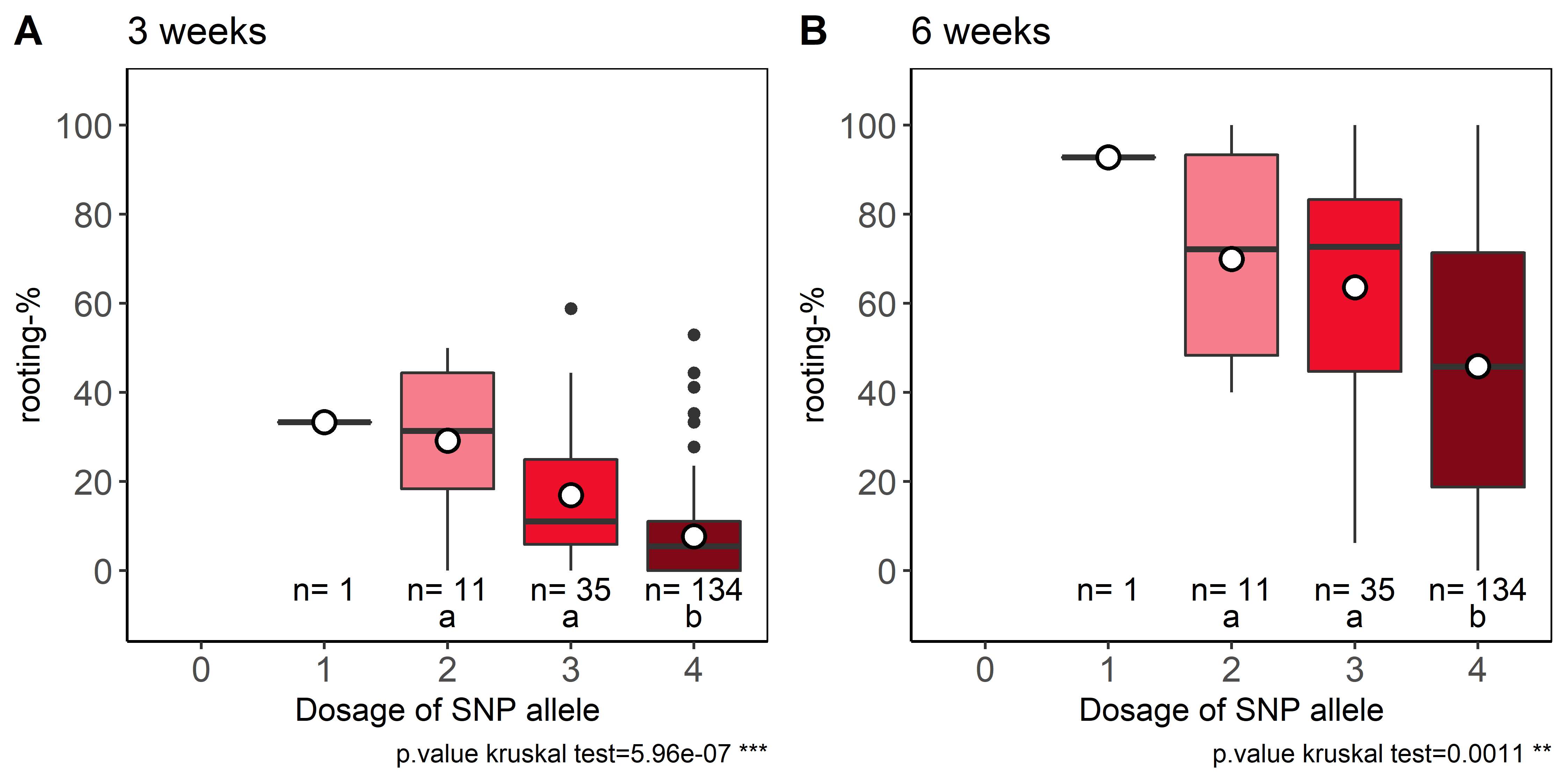

Supplement: S2 Fig — Allele dosage effects for the SNP RhK5_4872_1159Q on AR formation percentages after 3 (A) and 6 weeks (B) for 95 cut and 95 garden roses. X-axis values show the dosages for the SNP allele from nulliplex (0) to quadruplex (4), where the number of individuals per ADG is given by n. Letters indicate significance groups with respect to Fisher´s LSD criterion for p <0.05 under consideration of the Holm‒Bonferroni adjustment. (TIF) [file pone.0287452.s002.tif]

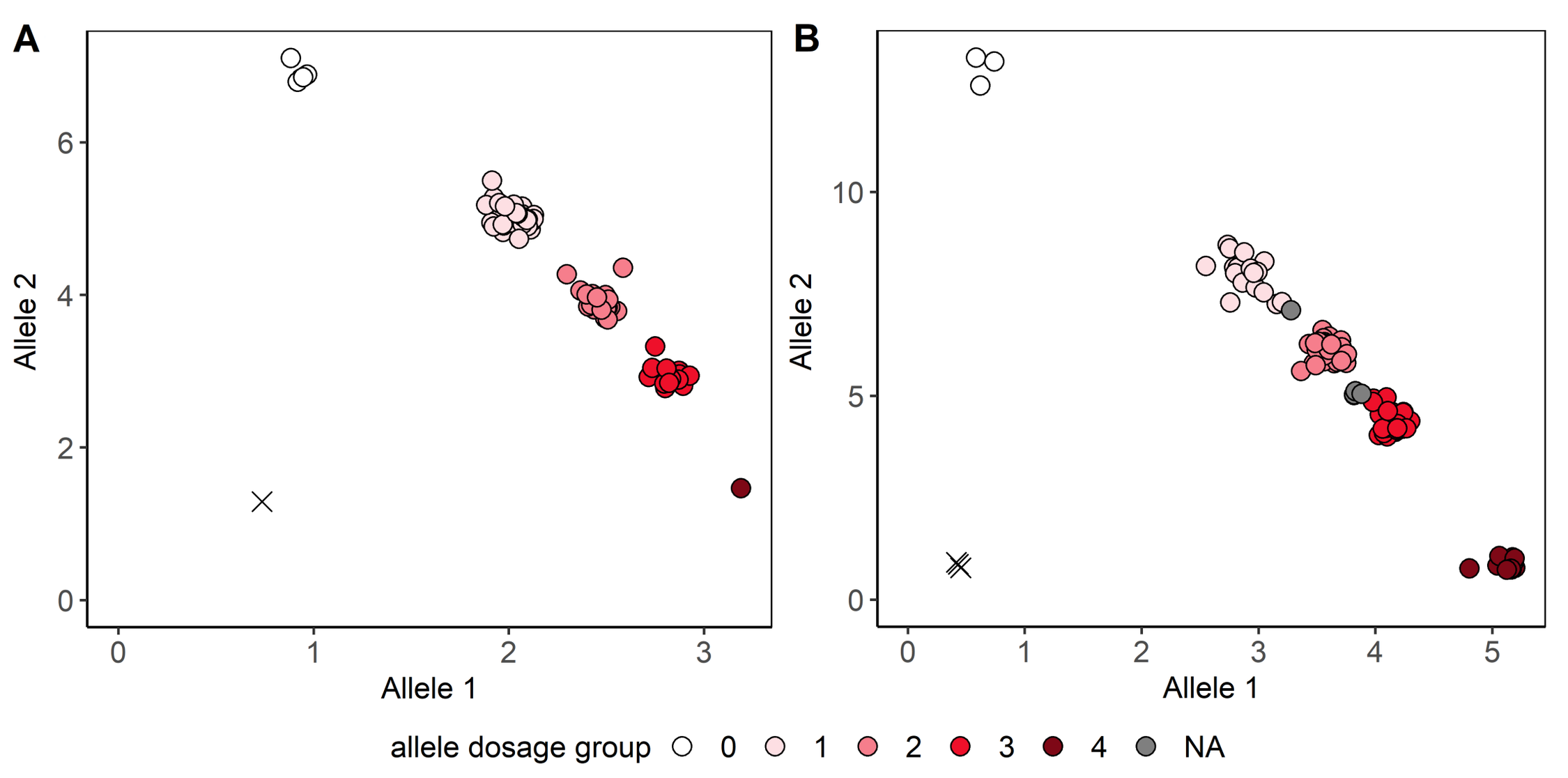

Supplement: S3 Fig — Genotyping results in the cut rose (A) and garden rose sets (B) with the KASP marker assigned to SNP RhK5_69_1627P. The X-axis indicates HEX fluorescence intensity (Allele 1), and the Y-axis indicates FAM fluorescence intensity (Allele 2). The white and darkest red dots represent the homozygous individuals, and blended colours represent the heterozygous individuals. Black crosses indicate the water control, and genotypes with undetermined allele dosages are displayed in grey. The ADGs were determined from the fluorescence signal ratios by using fitTetra [43]. (TIF) [file pone.0287452.s003.tif]

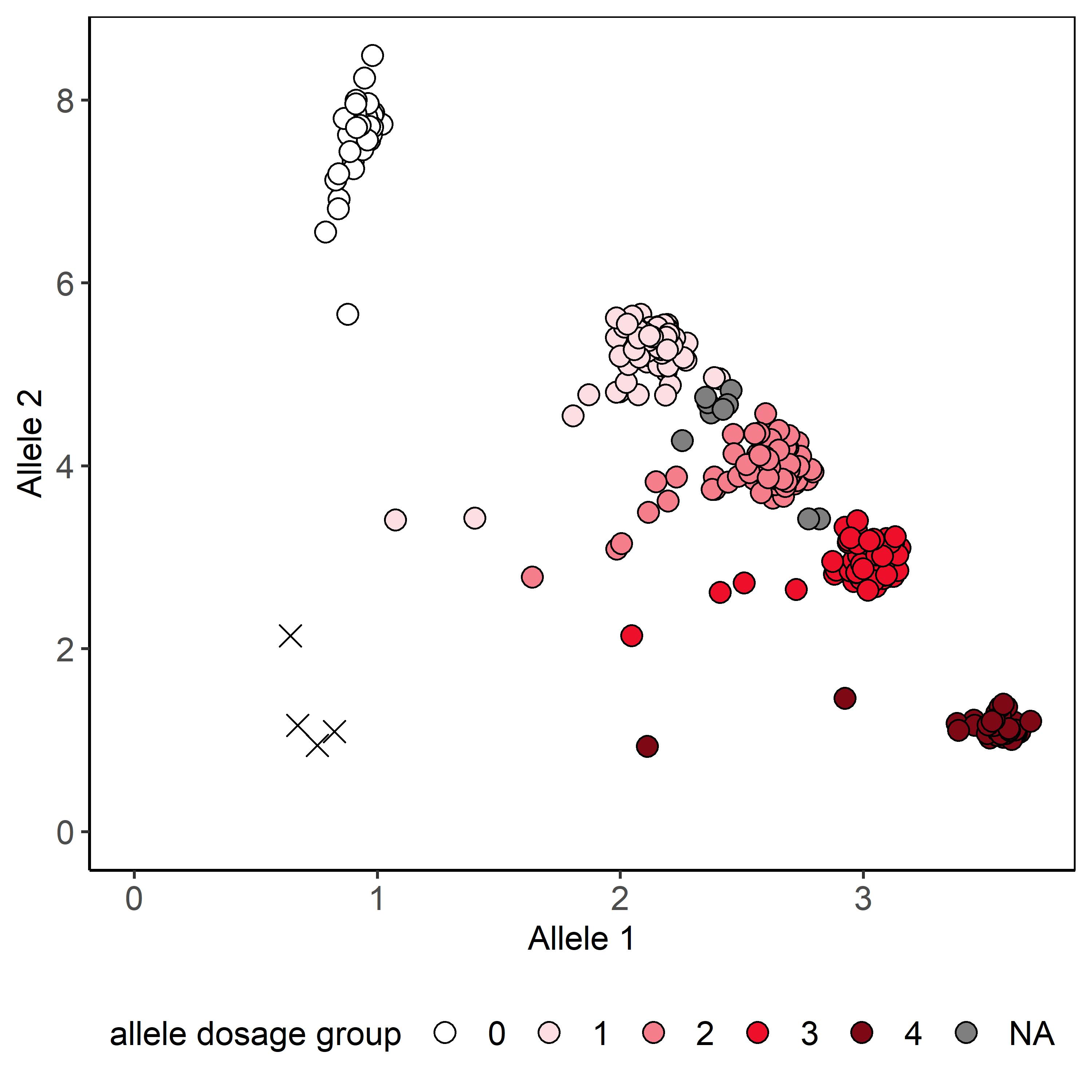

Supplement: S4 Fig — The X-axis indicates HEX fluorescence intensity (Allele 1), and the Y-axis indicates FAM fluorescence intensity (Allele 2). The white and darkest red dots represent the homozygous individuals, and the blended colours represent the heterozygous individuals. Black crosses indicate the water control, and genotypes with undetermined allele dosages are displayed in grey. The ADGs were determined from the fluorescence signal ratios by using fitTetra [43]. (TIF) [file pone.0287452.s004.tif]
